# Mobilization of Fluids in the Intensive Treatment of Primary and Secondary Lymphedemas

**DOI:** 10.1155/2018/6537253

**Published:** 2018-05-10

**Authors:** Jose Maria Pereira de Godoy, Henrique Jose Pereira de Godoy, Thatiany Gracino de Marqui, Luis Cesar Spessoto, Maria de Fatima Guerreiro Godoy

**Affiliations:** ^1^Cardiology and Cardiovascular Surgery Department, The Medicine School in São José do Rio Preto (FAMERP), CNPq (National Council for Research and Development), São José do Rio Preto, SP, Brazil; ^2^Universidade Federal do Mato Grosso Cuiabá, MT and Researcher Group of the Clínica Godoy, São José do Rio Preto, SP, Brazil; ^3^Medicine School of São Jose do Rio Preto (FAMERP), São José do Rio Preto, SP, Brazil; ^4^Medicine School in São José do Rio Preto (FAMERP) and Researcher Group of the Clínica Godoy, São Jose do Rio Preto, SP, Brazil

## Abstract

**Background:**

Lymphedema is a clinical condition resulting from the accumulation of macromolecules in the interstitial space with a consequent buildup of fluids.

**Aim:**

The objective of this study was to compare the therapeutic response to treatment that mobilizes fluids between primary and secondary lymphedemas.

**Method:**

Thirty-three patients with severe leg lymphedema who underwent intensive treatment for five consecutive days in 2013 and 2014 at the Clínica Godoy were evaluated in a prospective clinical trial. Diagnosis was based on the patient's history and physical examination. Treatment consisted of eight hours/day of Mechanical Lymphatic Therapy using an electromechanical device (RAGodoy®) that performs plantar flexion and extension associated with 15 minutes of Cervical Lymphatic Therapy, a technique developed by Godoy and Godoy that involves stimulation in the cervical region and a grosgrain compression stocking alternated with elastic bandages. The unpaired *t*-test and Fisher's exact test were used for statistical analysis with an alpha error of 5% (*p* value < 0.05) being considering acceptable. Secondary lymphedema was more prevalent in women (Fisher exact test *p* value < 0.01).

**Results:**

The age of patients with secondary lymphedema was greater than those with primary lymphedema (unpaired *t*-test: *p* value < 0.03). The mean volume losses were 64.62% and 48.35% for the patients with secondary and primary lymphedema, respectively (*p* value < 0.03).

**Conclusion:**

Women are more prevalent and older in the secondary lymphedema group. Volumetric reductions below the knee are faster with intensive treatment for secondary rather than for primary lymphedema.

## 1. Introduction

Lymphedema is a clinical condition resulting from the accumulation of macromolecules in the interstitial space with a consequent buildup of fluids [1, 2]. This condition can have primary or secondary causes. Patients suffering from primary lymphedema are born with some type of alteration of the lymphatic system and secondary cases are the result of damage to the lymphatic system during the lives of patients.

Diagnosis is usually based on a medical history and physical examination; however, in some cases, complementary examinations are required. A volumetric evaluation is necessary to quantify the edema and to evaluate whether the difference in limb size can be considered clinically significant [3]. In relation to the volume evolution, mild lymphedema is defined as a difference of volume <20% in relation to the contralateral limb, moderate with differences between 20 and 40% and above 40% considered severe. In relation to the clinical evolution, stage I is when the edema appears during the day and disappears at night, stage II is when the edema is constant without disappearing even after resting for one week, and stage III is when the limb has large deformities [2].

An association of therapeutic techniques, usually including manual or mechanical lymph drainage, compression therapy, and exercises, is recommended with the main aim being to improve the quality of life of patients [2, 4]. In recent years, a new concept of lymphatic drainage, Cervical Lymphatic Therapy (cervical stimulation), has been developed, which, as believed, stimulates the nervous system leading to drainage of the lymph [5]. An apparatus (RAGodoy) has also been developed that performs Mechanical Lymphatic Drainage [6]. Another contribution is the development of a stocking made of grosgrain fabric that can be used for upper and lower limbs. Grosgrain is inelastic but generates a significant resting pressure [7]. An intensive form of outpatient therapy (6–8 hours/day) has been proposed that can reduce about 50% of the lymphedema volume within five days [2]. However, a difference in response to treatment has been observed between primary and secondary lymphedema. The objective of this study was to compare the therapeutic response to treatment used to mobilize fluids between primary and secondary lymphedemas.

## 2. Methods

Thirty-three patients with severe leg lymphedema who underwent intensive treatment for five consecutive days in 2013 and 2014 at the Clínica Godoy, São Jose do Rio Preto, Brazil, were evaluated in a prospective clinical trial. The inclusion criteria were stage III lymphedema, that is, a volumetric difference between the legs of more than 40%. Patients were consecutively enrolled in this trial. Diagnosis was based on the patient's history and physical examination. Treatment consisted of eight hours/day of Mechanical Lymphatic Therapy using an electromechanical device (RAGodoy) that performs plantar flexion and extension, associated with 15 minutes of Cervical Lymphatic Therapy, a technique developed by Godoy and Godoy that involves stimulation in the cervical region and a grosgrain compression stocking alternated with elastic bandages. The patient's gender, age, and type of lymphedema (primary and secondary) were recorded and the volume loss during five days of treatment was evaluated by volumetry below the knee. The unpaired* t*-test and Fisher's exact test were used for statistical analysis with an alpha error of 5% (*p* value < 0.05) being considered acceptable. The study was approved by the Ethics Committee of the Medicine School of Sao Jose do Rio Preto (#078465/2016, CAAE: 58523216.5.0000 5415/2016).

## 3. Results

**Figure 1:**
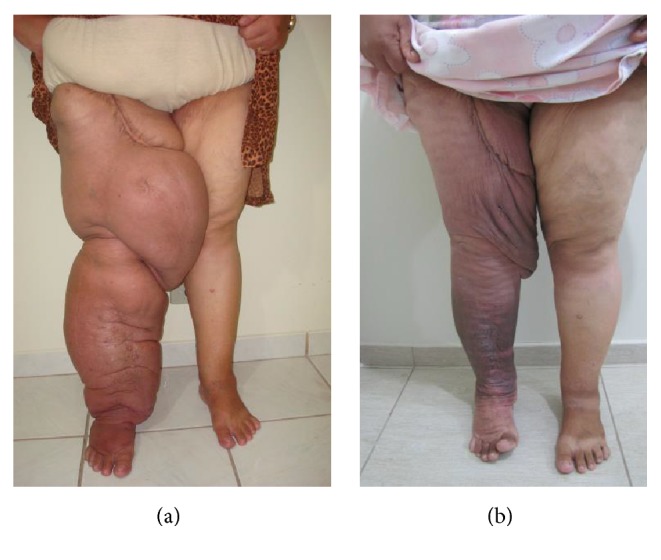
(a) and (b) illustrate initial treatment and treatment after 5 days in a secondary lymphedema.

**Figure 2:**
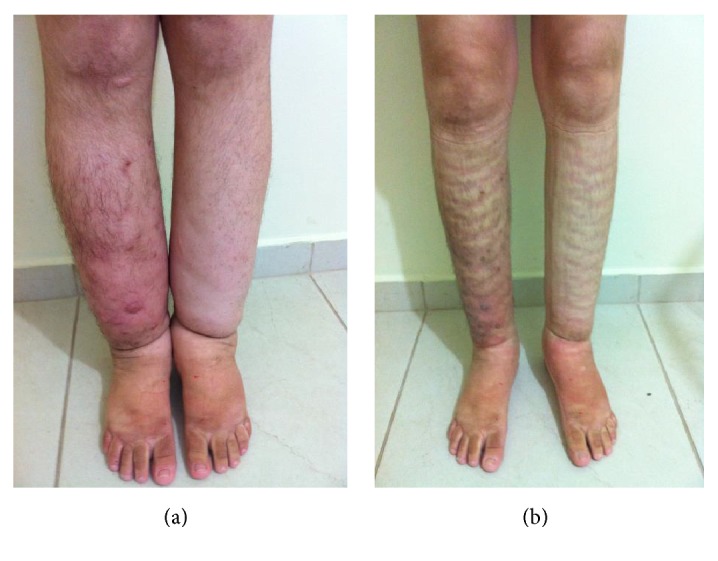
(a) and (b) show initial treatment and treatment after 5 days in primary lymphedema.

The ten patients with secondary lymphedema were all women with a mean age of 49.0 years. Of the 23 patients with primary lymphedema, ten were male and 13 were female with a mean age of 40.5 years. Secondary lymphedema was more prevalent in women (Fisher exact test *p* value < 0.01). The age of patients with secondary lymphedema was greater than those with primary lymphedema (unpaired *t*-test: *p* value < 0.03). The mean volume losses were 64.62% and 48.35% for the patients with secondary and primary lymphedema, respectively (*p* value < 0.03). Figures 1(a) and 1(b) illustrate initial treatment and treatment after 5 days in a secondary lymphedema and Figures 2(a) and 2(b) show initial treatment and treatment after 5 days in primary lymphedema.

## 4. Discussion

The present study shows that there is a difference in the percentages of volumetric losses of edema of the lower limbs between primary and secondary lymphedema in the first week of intensive treatment. Secondary lymphedema is a sequel of gynecological cancer treatment in all the patients with the mean age being significantly higher for this group compared to the primary lymphedema group. The literature shows that the prevalence of lymphedema after gynecological cancer treatment is significant and increases the discomfort of these patients [8, 9].

The intensive form of treatment allows a significant reduction, about 50% in volume, within five days; the changes are fast and it is easy to observe the reduction of the edema [2]. The therapy employed was an association (Mechanical Lymphatic Therapy, Cervical Lymphatic Therapy, and a compression stocking made of grosgrain). Mechanical Lymphatic Therapy, used for eight hours per day, primarily stimulates drainage below the knee and the stockings cause constraint of the entire leg.

The hypothesis for the difference in volume loss within the first week is that, in primary lymphedema, the edema usually begins at the feet with the progression to fibrosis being greater in the distal region. In secondary lymphedemas, however, the edema is more significant at the thigh and the feet are not always affected by fibrosis.

In primary lymphedema, the proximal region of the thigh is normalized faster than the distal edema with the last region to be normalized being the feet, more specifically the toes. The compression stocking that initially was needed for the entire leg is only needed for the lower leg with the evolution of therapy. In many patients, maintenance is achieved with a grosgrain stocking worn once a week with knee-length elastic stockings being worn on the other days.

In secondary lymphedema, the reduction of the edema at the thigh is slower than with primary lymphedema, while the decrease is faster below the knee. The stocking needs to provide constraint to the entire limb throughout treatment because the proximal thigh requires compression until the end of therapy. The same can be said about maintaining the results; after total reduction of the edema, as evaluated by bioimpedance and volumetry, stockings are necessary for the entire leg in most patients.

Although the evolution related to treatment is different for the two types of lymphedema, normalization can be achieved in both. During treatment, volume reductions are higher in the first week with around 10–30% being lost in the second. Normalization can be achieved in weeks or months depending on the intensity of the treatment.

Maintenance is essential for all patients with elastic stockings, after total normalization of edema, being a good option. It is noted that good results cannot be maintained before total normalization of the edema has been achieved. One soon-to-be-published study that evaluated 30/40 mmHg compression stockings identified that it was possible to maintain the reduction for at least four months after normalization. Another study being completed now shows that it is possible to maintain the results after normalization for a period longer than one year by associating a grosgrain stocking twice a week with elastic stockings on five days. Another important aspect is that the stockings need to be well adjusted to achieve the best results because of a positive synergistic effect [10].

## 5. Conclusion

Women are more prevalent and older in the secondary lymphedema group. Volumetric reductions below the knee are faster with intensive treatment in secondary rather than in primary lymphedema.

## References

[1] Lee B. B., Antignani P. L., Baroncelli T. A. (2015). IUA-ISVI consensus for diagnosis guideline of chronic lymphedema of the limbs.

[2] de Godoy J. M. P., de Godoy M. D. F. G. (2010). Godoy & Godoy technique in the treatment of lymphedema for under-privileged populations.

[3] Hassanein A. H., Maclellan R. A., Grant F. D., Greene A. K. (2017). Diagnostic Accuracy of Lymphoscintigraphy for Lymphedema and Analysis of False-Negative Tests.

[4] Huggenberger K., Wagner S., Lehmann S., Aeschlimann A., Amann-Vesti B., Angst F. (2015). Health and quality of life in patients with primary and secondary lymphedema of the lower extremity.

[5] de Godoy J. M. P., Godoy M. F. G. G., Meza M. C. (2008). Godoy & Godoy technique of cervical stimulation in the reduction of edema of the face after cancer treatment.

[6] Siqueira K. D. S. (2009). Mariângela Grochoski Karan. Volumetric alterations utilizing the RAGodoy® device to treat lymphedema of the lower extremities.

[7] de Godoy J. M. P., de Godoy A. C. P., Godoy M. F. G. (2015). Godoy Ana Carolina Pereira de, Godoy Maria de Fatima Guerreiro. Godoy Godoy Compression Sleeve in the Treatment of Arm Lymphedema: New Concepts for Materials. http://dx.doi.org/10.1590/S1516-89132015060286.

[8] Bae H. S., Lim M. C., Lee J. S. (2016). Postoperative lower extremity edema in patients with primary endometrial cancer.

[9] Hareyama H., Hada K., Goto K. (2015). Prevalence, classification, and risk factors for postoperative lower extremity lymphedema in women with gynecologic malignancies.

[10] de Godoy J. M. P., Lopes Pinto R., Pereira De Godoy A. C., De Fátima Guerreiro Godoy M. (2014). Synergistic effect of adjustments of elastic stockings to maintain reduction in leg volume after mechanical lymph drainage.

